# Assessment of asymptomatic bacteriuria and sterile pyuria among antenatal attendants in hospitals in northern Ghana

**DOI:** 10.1186/s12884-020-02936-6

**Published:** 2020-04-22

**Authors:** Akosua Bonsu Karikari, Courage Kosi Setsoafia Saba, David Yembilla Yamik

**Affiliations:** 1 Department of Clinical Microbiology, School of Medicine and Health Sciences, University for Development Studies, P. O. Box TL 1350, Tamale, Ghana; 2 Department of Biotechnology, Faculty of Agriculture, University for Development Studies, P. O. Box TL 1882, Tamale, Ghana

**Keywords:** Asymptomatic bacteriuria, Sterile pyuria, Antibiotic susceptibility, Tamale, Ghana

## Abstract

**Background:**

Asymptomatic bacteriuria (ASB) and sterile pyuria (SP) are complexities of UTI whose prevalence are not known in the northern sector of Ghana. Our aim was to determine the occurrence of sterile pyuria and asymptomatic bacteriuria among pregnant women accessing antenatal care at a secondary and tertiary care hospitals in Tamale, northern Ghana.

**Methods:**

A cross sectional study was conducted by screening 530 pregnant women with no signs of acute urinary tract infection attending antenatal clinic for a period of 6 months. Midstream urine was collected for microscopy, quantitative urine culture and antibiotic susceptibility testing. Data analysis was carried out using the Statistical Package for Social Sciences version 20.

**Results:**

Asymptomatic bacteriuria was respectively 20 and 35.5% at Tamale Central and Tamale Teaching Hospital out of the 390 and 90 women screened. Sterile pyuria was found among 66% of the 50 women presenting at Tamale Central Hospital. More than 64% of isolates recovered from ASB patients were *S. aureus* and *coagulase negative Staph.* (*CoNS*). *Escherichia coli* was the dominant species among members of the enterobacteriaceae isolated. Highest susceptibility was recorded against gentamicin and amikacin while most resistance was to Ampicillin, cotrimoxazole, chloramphenicol and nitrofurantoin. Resistance to imipenem and vancomycin were 28.8 and 52%, with strains showing multiple drug resistance of between 81 and 92%.

**Conclusion:**

The prevalence of asymptomatic bacteriuria is appreciably higher (20–35.5%) than documented rates in the southern sector of the country. The presence of sterile pyuria which may be an indication of asymptomatic renal impairment and most often overlooked in antenatal management is 66%. Empirical treatment of UTIs at the Tamale Central and Teaching Hospital without confirmation of susceptibility may result in treatment failure. It is necessary to screen and treat pregnant women for ASB and SP due to the complications associated with these conditions.

## Background

Urinary Tract Infection (UTI) is the invasion of microbes and the ensuant proliferation on part or the entire urinary tract [1]. It is the most common disorder caused by bacterial agents in pregnancy, which may lead to complications in neonates of such mothers in case of inappropriate diagnosis and treatment. As one of the most frequent acquired infections, UTI has an evident role in raising the number of stillbirth deliveries [2, 3]. Yearly, UTI and its related complications are the cause of nearly 150 million deaths worldwide [4]. Urinary tract infections in pregnancy is categorised into symptomatic and asymptomatic infections [5]. Asymptomatic bacteriuria is the commonest cause of UTI in pregnancy and mainly involves the lower urinary tract while the upper urinary tract engagement may result in symptomatic bacteriuria characterised by pyelonephritis [6].

Asymptomatic bacteriuria (ASB) is described as a significant bacteriuria without symptoms of UTI. Pregnant women with ASB have a higher risk to deliver premature or low-birth weight infants, develop pre-eclampsia and polyhydramnios [7–9]. Other conditions associated with ASB include transient renal failure, acute respiratory distress syndrome, shock and haematological abnormalities which occur in untreated or inadequately managed cases [9, 10]. Urinary tract infections and pyelonephritis in pregnancy has also been linked to morbidity in both mother and foetus [8]. Culturable bacteria species usually recovered from ASB infections fall under fourteen genera although non-culturable pathogens have also been implicated [11]. The Enterobacteriaceae are responsible for nearly (90%) all cases of asymptomatic bacteriuria with *E. coli* being dominant. *Enterococcus* spp., *Staphylococcus aureus* and coagulase-negative *Staphylococci* may also cause ASB [12, 13].

The Infectious Diseases Society of America Guidelines for the diagnosis and treatment of asymptomatic bacteriuria in adults (IDSA) recommends that all pregnant women should be screened for bacteriuria by urine culture at least once in early pregnancy, and if results are positive, treatment is justified. Screening for and treatment of asymptomatic bacteriuria is necessary for patients undergoing transurethral resection of the prostate and other urologic procedures in which mucosal bleeding is expected. However no reference can be made for screening for or treatment of asymptomatic bacteriuria in renal transplant or other solid organ transplant recipients [13, 14].

Another urinary tract condition which is also not rare in pregnancy is Sterile Pyuria (SP). Although no current definition exists [15], SP is considered if a mid-stream urine specimen has 10 or more white blood cells per cubic millimeter or a urinary dipstick test is leukocyte esterase positive with no associated positive urinary culture [16]. Sterile pyuria has vast aetiological spectrum including sexually transmitted diseases, tuberculosis, interstitial cystitis, chlamydia and cystitis [16]. Population-based studies recount it as a highly prevalent condition with 13.9% of women and 2.6% of men being affected [17], with its preponderance in women attributable to pelvic infections [18].

Various studies have consistently revealed that early diagnosis and treatment of UTI in pregnancy exorbitantly reduce sequelae associated with the condition. Treatment of ASB in pregnancy with antibiotics has been found to decrease pyelonephritis from 35 to 4% [19], improve fetal outcomes and prevent preterm deliveries. Several institutions prescribe screening of pregnant women for ASB due to the benefits of treatment [20, 21]. But, in many developing countries such as Ghana, ASB screening in pregnancy is normally overlooked in antenatal management. The prevalence of asymptomatic bacteriuria and sterile pyuria in the Northern regions of Ghana are not known. The aim of this assessment was to screen mid-stream urine of antenatal attendants at the Tamale Teaching Hospital and Tamale Central Hospital for sterile pyuria and asymptomatic bacteriuria with associated agents and their susceptibility profiles. This was to contribute to the literature bank of urinary tract infections at these secondary and tertiary care hospitals.

## Methods

### Study design

A cross sectional study was conducted from 16th April 2018 to 10th September 2018 at the Tamale Teaching Hospital (TTH) and Tamale Central hospital (TCH). The Tamale Teaching Hospital is an 800 bed capacity tertiary care facility which provides referral services to three Regions in the northern sector of Ghana and affiliated to the Medical School of the University for Development Studies. The Tamale Central Hospital is a secondary care facility which supports the teaching hospital in providing healthcare services to the populace in Tamale and its environs.

### Recruitment of study participants

A total of 530 pregnant women of all ages attending these hospitals for antenatal care within the study period were recruited. Inclusion criterion for selection involved a pregnant woman visiting the outpatient clinic for routine checkup. Informed consent was sought from all the pregnant women before enrollment. Attendees who refused consent were excluded from the study. Socio-demographic information and diagnosis were obtained from the laboratory report form and their medical folders.

### Specimen collection and processing

Pregnant women were provided with sterile urine containers and tutored on how to collect midstream urine. Approximately 10mls of urine were collected from each participant. The urine samples were transported to the Spanish laboratory of the University for Development Studies for processing and analysis, within 4 h of collection.

### Urine microscopy

About 5 mL of adequately mixed urine sample was centrifuged at 3000 rpm for 10 min. A drop of the sediment was placed on a glass slide, cover slipped and examined under the microscope to detect pus cells, red blood cells, casts and crystals. The presence of 10 or more pus cells/high-power field (HPF) was indicative of pyuria. Sterile pyuria in this study was defined as specimens scoring 10 or more pus cells but recorded no growth on Cysteine Lactose Electrolyte Deficient agar (CLED) plates after adequate incubation period of 24 h.

### Urine culture and identification

Urine samples were cultured on CLED using a standardized (0.01 mL) wire loop. The plates were incubated at 37 °C and read after 24 h for significant bacteriuria. Significant bacteriuria was defined as a quantitative count of ≥10^5^CFU/ml. Pathogens were identified using standardized biochemical tests, Mannitol salt agar and sugar fermentation using Triple Sugar Iron agar from 24 h pure culture colonies. Asymptomatic bacteriuria in this study was considered when the bacterial value was ≥10^5^ but participants had no symptoms of acute urinary tract infections.

### Antibiotic susceptibility test

Antibiotic susceptibility tests were conducted using Kirby Bauer disc diffusion method. Mueller-Hinton agar plates were inoculated with 0.5 McFarland standard saline suspension and incubated at 37 °C for 24 h [22]. The antibiotics analysed included, ciprofloxacin 10 μg, gentamicin 10 μg, erythromycin 15 μg, ceftriaxone 30 μg, chloramphenicol 30 μg, nitrofurantoin 50 μg, tetracycline 30 μg, ampicillin 10 μg, clindamycin 10 μg, vancomycin 30 μg, cefoxitin 30 μg, amikacin 30 μg, trimethoprim-sulfamethoxazole 25 μg, imipenem 10 μg, amoxacillin clavulanic acid 30 μg and norfloxacin 10 μg. The recorded inhibition zones were interpreted using CLSI breakpoints. Quality control strains of *Escherichia coli* (ATCC 25922) and *Staphylococcus aureus* (ATCC 25923) were used. Multidrug resistance in this study was defined as resistance of isolates to three (3) or more classes of antibiotics.

### Data management

Collected data was analysed by descriptive statistics using frequencies and percentages using IMB SPSS version 20. Results were presented in tables. Associations between categorical outcome variables were conducted using the Pearson- Chi square test at the 95% confidence level. A two tailed *p*-value of < 0.05 was considered statistically significant.

### Ethical consideration

Ethics approval was obtained from the Ethical Review Committee of the Tamale Teaching Hospital (TTHERC/25/06/19/14). Verbal informed consent was sought from the pregnant women after sufficient information regarding the study was provided. This is because while some of the women were unable to read and write others felt reluctant to write and saw the process as a bother.

## Results

Asymptomatic bacteriuria was present among 20 and 35.5% of pregnant women seeking antenatal care at the Tamale Central Hospital and Tamale Teaching Hospital respectively. More than 60% of these women were between the ages of 20-29 years but less frequent among ages less than 20 years and more than 40 year groups. The difference in prevalence at these hospitals was not significant, *p* > 0.05, Table 1.

**Table 1:** Age distribution of women with asymptomatic bacteriuria

	Tamale Central Hospital	Tamale Teaching Hospital	
	*N* = 390	*N* = 90	
Age/yrs.	Frequency (%)	Frequency (%)	*P*-value
< 20	3 (3.8)	1 (3.1)	0.157
20–29	50 (64.1)	21 (65.6)	
30–39	23 (29.5)	10 (31.3)	
40–49	2 (2.6)	0	
Total	78 (20)	32 (35.5)	

Organisms isolated from pregnant women with ASB at the Tamale Central and Teaching hospitals were similar and there was no significant difference in the rate of pathogen recovery (*p* = 0.423). At both hospitals, Gram positives mainly *S. aureus* and coagulase negative Staph. (C_O_NS) were the dominant species identified as 64.1 and 75.0% were respectively found at Central and the Teaching hospital. These pathogens were rife among age group 20–29 with a rate of 42.3% at Central and 50% at Teaching hospital. This was followed by age group 30–39 with respective prevalence of 17.9 and 25%. *Escherichia coli* was more commonly isolated among the Gram negatives, followed by *Enterobacter* and *Klebsiella sp.*, Table 2.

**Table 2:** Recovered isolates from pregnant women presenting with asymptomatic bacteriuria

		Tamale Central Hospital							
		Gram Positives			Gram Negatives				
Age/Yrs	Frequency (%)	*S. aureus*	*CoNS*	*Strept.*	*E. coli*	*Kleb.sp*	*Entero.*	*Serratia*	*Pseud.*
< 20	3 (3.8)	2	1	0	0	0	0	0	0
20–29	50 (64.1)	14	19	1	6	4	4	2	0
30–39	23 (29.5)	7	7	2	5	0	0	0	0
40–49	2 (2.6)	0	0	0	0	1	6	0	1
Total	78 (100)	23	27	3	11	4	7	2	1
		Tamale Teaching Hospital							
		Gram Positives			Gram Negatives				
Age/Yrs	Frequency (%)	*S. aureus*	*CoNS*	*Strept.*	*E. coli*	*Kleb.sp*	*Entero.*	*Serratia*	
< 20	1 (3.1)	0	0	1	0	0	0	0	
20–29	21 (65.6)	5	11	0	1	1	1	2	
30–39	10 (31.3)	6	2	0	0	1	1	0	
40–49	0	0	0	0	0	0	0	0	
Total	32 (100)	11	13	1	1	2	2	2	

*Strept.* Streptococcus sp, *Kleb.* klebsiella sp, *Entero.* Enterobacter sp, *pseud.* pseudomonas sp, *CoNS* coagulase negative Staphylococcus

Generally resistance was commonly observed among the Gram negatives. The isolates were fairly susceptible to gentamicin and amikacin as resistance of below 20% was recorded at both hospitals. Resistance to the fluoroquinolones ranged from 0 to 26.6% to ciprofloxacin and 12–42.9% to norfloxacin. Among the broad spectrum antibiotics, isolates showed highest resistance to Ampicillin (76–96%) followed by erythromycin (32–85.7%), tetracycline (30–76%), chloramphenicol (39–71.4%) and amoxicillin clavulanic acid (28–57%). Resistance to nitrofurantoin ranged from 33 to 71% and between 43 and 71% was recorded against trimethoprim sulphamethoxazole. Isolates resistance to ceftriaxone was less than 50% (22–42.9%). About 41–52% of the Gram positives showed resistance to vancomycin as Gram negative resistance to imipenem ranged from 8 to 28.6%, Tables 3 and 4. Isolates from Central Hospital showed less resistance to ceftriaxone (22–28%) and amoxicillin clavulanic acid (28–39.6%) as against 32–42.9% and 52–57.1% respectively recorded at the Tamale Teaching Hospital. But the Central Hospital strains showed higher resistance (30–76%) to tetracycline than isolates from Tamale Teaching Hospital (28–32%). The difference in resistance rates in the two hospitals was found to be statistically significant, *p* = 0.000. Multidrug resistance of 92.3 and 81.3% were recorded among isolates recovered respectively from Central and the Teaching hospital, Table 5.

**Table 3:** Susceptibility profile of isolates recovered from pregnant women with asymptomatic bacteriuria

	Tamale Central Hospital						Tamale Teaching Hospital					
	*N* = 53			*N* = 25			*N* = 25			*N* = 7		
	Gram Positive			Gram Negative			Gram Positive			Gram Negative		
Antibiotic	S	I	R	S	I	R	S	I	R	S	I	R
Ciprofloxacin	41	4	8	18	1	6	25	0	0	5	0	2
Cotrimoxazole	27	3	23	8	2	15	13	1	11	2	0	5
Gentamicin	42	5	6	23	0	2	23	2	0	6	0	1
Amikacin	44	3	6	21	0	13	21	1	3	6	0	1
Ampicillin	5	0	48	4	2	19	1	0	24	1	0	6
Amoxicillin/clav.	31	1	21	12	6	7	12	0	13	2	1	4
Chloramphenicol	30	2	21	10	0	15	13	0	12	1	1	5
Nitrofurantoin	24	11	18	5	3	17	12	2	11	2	0	5
Ceftriaxone	13	28	12	12	6	7	5	12	8	2	2	3
Erythromycin	14	13	26	3	3	19	8	9	8	1	0	6
Tetracycline	31	6	16	5	1	19	15	2	8	4	1	2
Norfloxacin	34	1	18	18	0	7	22	0	3	4	0	3
Cefoxitin	34	8	11	NT			12	2	11	NT		
Clindamycin	21	4	28	NT			9	2	14	NT		
Vancomycin	30	1	22	NT			12	0	13	NT		
Imipenem	NT			23	0	2	NT			5	0	2

*NT* Not tested, Cotrimoxazole- SXT, *S* sensitive, *I* intermediate, *R* resistant

**Table 4:** Resistance patterns of recovered isolates from pregnant women with ASB

	Tamale Central Hospital		Tamale Teaching Hospital	
	Resistance %			
Antibiotic	Gram positive	Gram negative	Gram positive	Gram negative
Ciprofloxacin	15.1	24	0	26.6
Co-trimoxazole	43.4	60	44	71.4
Gentamicin	11.3	8	0	14.3
Amikacin	11.3	12	12	14.3
Ampicillin	90.6	76	96	85.7
Amoxicillin/clav.	39.6	28	52	57.1
Chloramphenicol	39.6	60	48	71.4
Nitrofurantoin	33.9	68	44	71.4
Ceftriaxone	22.6	28	32	42.9
Erythromycin	49.1	76	32	85.7
Tetracycline	30.2	76	32	28.6
Norfloxacin	33.9	28	12	42.9
Cefoxitin	20.8	NT	44	NT
Clindamycin	52.8	NT	56	NT
Vancomycin	41.5	NT	52	NT
Imipenem	NT	8	NT	28.6

*NT* Not tested

**Table 5:** Multidrug resistance exhibited by isolates

	Tamale Central Hospital		Tamale Teaching Hospital	
Isolates	No. of isolates	MDR (%)	No. of isolates	MDR (%)
*S. aureus*	23	23(100.0)	11	11(100.0)
*CONS*	27	22(81.5)	13	8(61.5)
*Streptococcus sp.*	3	2(66.7)	1	1(100.0)
*E. coli*	11	11(100.0)	1	1(100.0)
*Klebsiella sp.*	4	4(100.0)	2	2(100.0)
*Enterobacter sp.*	7	7(100.0)	2	2(100.0)
*Serratia*	2	2(100.0)	2	1(50.0)
*Pseudomonas sp.*	1	1(100.0)	0	0
Total	78	72(92.3)	32	26(81.3)
Gram positives	53	47 (88.7)	25	20(80)
Gram negatives	25	25 (100.0)	7	6(85.7)

Of the 50 attendants screened for sterile pyuria at the Tamale Central Hospital, 66% were positive with more than 70% of the women within the ages of 20–29 years and a mean age of 25.9 was recorded, Table 6.

**Table 6:** Sterile pyuria among 50 pregnant women at Tamale Central Hospital

	*N* = 50	
Age/yrs.	Frequency	%
< 20	3	9.1
20–29	24	72.7
30–39	5	15.2
40–49	1	3
Total	33 (66%)	100

## Discussion

The study revealed prevalence of asymptomatic bacteriuria among pregnant women seeking antenatal care at Tamale Central Hospital and Tamale Teaching Hospital at 20 and 35.5%; a rate much higher than 5.5 and 7.3% respectively reported from Korle-Bu Teaching Hospital in Accra and the Komfo Anokye Teaching Hospital in Kumasi both in the southern sector of Ghana [23, 24]. Other similar studies have shown varying prevalence rates; 10% in Egypt [25]; 4–7% in Canada [26]; 13.3% in Uganda [27] and 7% in Ethiopia [28]. But comparable to our finding is the 28.8% documented in Ibadan Nigeria although higher rates of 63.3 and 86.6% have been recorded in two studies in the same country [29, 30]. Disparities in ASB prevalence within and between countries are assignable to sexual contact, socioeconomic levels, cultural and religious behaviours concomitant to personal hygiene [25]. In Ghana, socioeconomic levels and cultural practices differ from the south to the north and this could have accounted for the sharp difference in prevalence.

Asymptomatic bacteriuria seem to be predominant in women aged 20 and 30 years and less in age group < 20 years [25, 31]. This was in agreement with our results and others reported in other sectors of the country [23, 24]. The explanation to the susceptibility of these age groups could be early and intensive sexual intercourse which may result in minor urethral trauma and transfer organisms from the perineum into the bladder [32].

Contrary to most studies which have Gram negatives especially *E. coli* as the frequently isolated bacteria, this investigation found Gram positive *S. aureus* and coagulase negative *Staphylococcus* (CoNS) as the commonly recovered pathogens at both hospitals. Among the isolated Gram negatives, *E. coli* was dominant followed by *Enterobacter* sp. and *Klebsiella* sp. This could be imputed to the fact that CoNS and *S. aureus* are normally encountered in UTIs in the 20–30 year group and more than 64% of our study population came from this group. In Ghana, Gram positive bacteria appears to be conventional in pregnant women presenting with ASB as *S. aureus* and *Enterococcus* dominated in similar analysis in the country [24, 33].

Susceptibility of isolates to gentamicin and amikacin were highest which could possibly be the infrequent prescription of these drugs in the treatment of UTI infections at both hospitals as these drugs are only used in the management of severe and vulnerable cases (Personal communication). Low susceptibilities were found against ampicillin, cotrimoxazole, chloramphenicol, erythromycin and nitrofurantoin. Antibiotic pressure resulting from persistent use and abuse could have accounted for the high resistance levels to these drugs. A worrying observation was the 28.8 and 52% resistance recorded against imipenem and vancomycin as these drugs (carbapenem and glycopeptides) used to be highly effective against members of the enterobacteriaceae and gram positives and assessed as the last line of treatment drugs in managing difficult to treat MDR pathogens in these hospitals. Considering these outcomes empirical treatment will not be safe without laboratory confirmation of the susceptibility of all classes of antibiotics.

Sterile pyuria is a pervasive condition in both primary and secondary care settings but limited literature exist to provide estimated prevalence in the community or hospital setting. This situation has led to inconsistent management ranging from absolute neglect of the finding to over investigation and unwarranted referrals [34]. At the Tamale Central hospital (secondary care facility), 66% of the 50 pregnant women screened had sterile pyuria which relates to the study of Shipman et al. [35] who reported 74% in women presenting to an emergency department in the United States.

This study had some limitations which are that, detailed information such as gestational ages of pregnancy, sexual activity which is considered a risk factor for developing UTI in women could not be captured. This is because most women in the study area do not feel comfortable giving out information on such details. Any attempt to persuade them normally led to their withdrawal from the study. But this lapse did not affect the purpose of the study which was to establish the prevalence of asymptomatic bacteriuria and sterile pyuria among pregnant women in secondary and tertiary care facility.

## Conclusion

The prevalence of asymptomatic bacteriuria at the Tamale Central and the Tamale Teaching Hospital is 20 and 35.5% which is considerably higher than what has been found in the southern sector of Ghana. *Staphylococcus aureus* and *coagulase negative Staph.* are the commonest causative organisms. Due to the complications associated with asymptomatic bacteriuria, the study recommends that screening is done for all pregnant women, and the appropriate antibiotics prescribed for treatment once culture and sensitivities are known. Sterile pyuria; a common but neglected entity was frequently encountered (66%) which may be a presentation of asymptomatic renal disease and therefore must be considered in managing pregnant women in these facilities.

## Data Availability

We consider our data private but the corresponding author will make it available upon reasonable request.

## Abbreviations

ASB: Asymptomatic bacteriuria
CLED: Cysteine lactose electrolyte deficient agar
CFU: Colony forming unit
TCH: Tamale Central Hospital
TTH: Tamale Teaching Hospital
CoNS: Coagulase negative *Staphylococcus*
MDR: Multidrug resistance
SP: Sterile pyuria
RPM: Revolution per minute
